# Case report: Acute HHV6B encephalitis/myelitis post CAR-T cell therapy in patients with relapsed/refractory aggressive B-cell lymphoma

**DOI:** 10.3389/fneur.2024.1334000

**Published:** 2024-02-29

**Authors:** Ningwen Li, Ruoxuan Zhang, Jue Wang, Xiaojian Zhu, Fankai Meng, Yang Cao, Gaoxiang Wang, Yang Yang

**Affiliations:** ^1^Department of Hematology, Tongji Hospital, Tongji Medical College, Huazhong University of Science and Technology, Wuhan, Hubei, China; ^2^Immunotherapy Research Center for Hematologic Diseases of Hubei Province, Wuhan, Hubei, China

**Keywords:** HHV6B, CAR-T cell therapy, encephalitis, myelitis, mNGS

## Abstract

**Background:**

The development of chimeric antigen receptor (CAR)-T cell therapy has revolutionized treatment outcomes in patients with lymphoid malignancies. However, several studies have reported a relatively high rate of infection in adult patients following CD19-targeting CAR T-cell therapy, particularly in the first 28 days. Notably, acute human herpesvirus 6 B (HHV6B) reactivation occurs in up to two-thirds of allogeneic hematopoietic stem cell transplantation patients.

**Case presentations:**

Herein, we describe a report of HHV6B encephalitis/myelitis in three patients with relapsed/refractory diffuse large B-cell lymphoma post CAR T-cell therapy. All three patients received multiple lines of prior treatment (range: 2–9 lines). All patients presented with fever that persisted for at least 2 weeks after CAR-T cell infusion (CTI). Both the onset time and duration were similar to those of the cytokine release syndrome (CRS); nevertheless, the CRS grades of the patients were low (grade 1 or 2). Delirium and memory loss after CTI were the earliest notable mental presentations. Neurological manifestations progressed rapidly, with patients experiencing varying degrees of impaired consciousness, seizures, and coma. Back pain, lumbago, lower limb weakness and uroschesis were also observed in Patient 3, indicating myelitis. High HHV6B loads were detected in all Cerebral spinal fluid (CSF) samples using metagenomic next-generation sequencing (mNGS). Only one patient required high-activity antivirals and IgG intravenous pulse treatment finally recovered, whereas the other two patients died from HHV6B encephalitis.

**Conclusion:**

Considering its fatal potential, HHV6B encephalitis/myelitis should be urgently diagnosed post CAR-T cell-based therapy. Furthermore, hematologists should differentially diagnose these conditions from CRS or other immunotherapy-related neurotoxicities as early as possible. The results of this study demonstrate the potential of mNGS in the early diagnosis of HHV6B infection, particularly when the organism is difficult to culture.

## Introduction

The introduction of chimeric antigen receptor (CAR)-T cell therapy has rapidly transformed the treatment landscape for lymphoid malignancies. In patients with relapsed or refractory disease who previously had limited treatment options, CAR-T cell therapy has shown impressive responses, including complete responses (CRs) in approximately 80% of patients with acute lymphoblastic leukemia (ALL) and 40–60% of those with aggressive lymphomas (1–4). The results of our serial clinical trials indicate that sequential infusion of CAR19/22-T cell was safe and efficacious in treating patients with R/R B-cell lymphomas (5, 6).

Importantly, several studies have reported that because of extensive prior antitumor therapies, the use of lymphodepleting chemotherapy, and severe therapy-associated toxicities, such as cytokine release syndrome (CRS) and B-cell aplasia, adult patients receiving CD19-targeting CAR-T cell therapy exhibit a high rate of infection, particularly in the first 28 days (7–9). Most early infections are bacterial, occurring in approximately 16.5% of patients, whereas respiratory viral infections predominate at later time points (7). Reactivation of Epstein–Barr virus (EBV) and cytomegalovirus (CMV) is the most frequent (7). However, two cases of human herpesvirus 6 B (HHV6B) encephalitis (10) and one case of HHV6B myelitis after CAR T cell therapy (11) have been previously reported.

HHV6 is a member of the β-herpesvirus subfamily and comprises two species, HHV6A and HHV6B (12). HHV6 infects approximately 90% of the population by 2 years of age, manifesting as Roseola infantum, and then remains dormant in the host with possible reactivation during immunosuppression (12). HHV6 reactivates in up to two-thirds of allogeneic hematopoietic stem cell transplant (HSCT) patients, typically within 3 weeks post-transplantation, and encephalitis develops in only a small proportion of patients experiencing HHV6 reactivation (13).

Human herpesvirus 6 (HHV-6), particularly the HHV-6B strain, is a major cause of encephalitis and other complications following allogeneic hematopoietic stem cell transplantation (HSCT) (14). Clinically, HHV-6 encephalitis typically presents 2–6 weeks after HSCT with symptoms of confusion, memory loss, seizures and insomnia. Diagnosis involves the detection of HHV-6B DNA in the cerebrospinal fluid, where mild protein elevation and lymphocytic pleocytosis are also noted. See below for notes on HHV6 viral load detection in CSF as it relates to encephalitis. Brain Magnetic Resonance Imaging (MRI) may initially appear normal, but may later show hyperintense lesions and abnormalities in the limbic region, particularly in the medial temporal lobes, indicating inflammation. Diagnosis is based on patient history, PCR for HHV-6 DNA and other tests, with chromosomally integrated HHV-6 (CIHHV-6) complicating the diagnosis due to the presence of inherited HHV-6 DNA (15). Risk factors include HHV-6 reactivation and poor T-cell function, with prognosis ranging from complete recovery to persistent neurological problems or death (16). Treatment includes antiviral drugs such as ganciclovir and foscarnet, and new strategies such as adoptive immunotherapy. Treatment is usually given for 3 weeks or until HHV-6 DNA is cleared. Prevention strategies are not well established and HHV-6B is also associated with other post-HSCT conditions such as myelosuppression, although evidence is limited (15). Overall, HHV-6B is a significant cause of infectious encephalitis after HSCT and improved diagnosis and treatment are needed, although there are few reports of HHV-6B encephalitis following CAR-T treatment (10, 11).

Herein, we report three cases of relapsed/refractory diffuse large B-cell lymphoma R/R with HHV6 early reactivation involving the central nervous system after CD19/CD22 CAR-T cell cocktail therapy at our institution between 2019 and 2020.

## Case report

**Figure 1:**
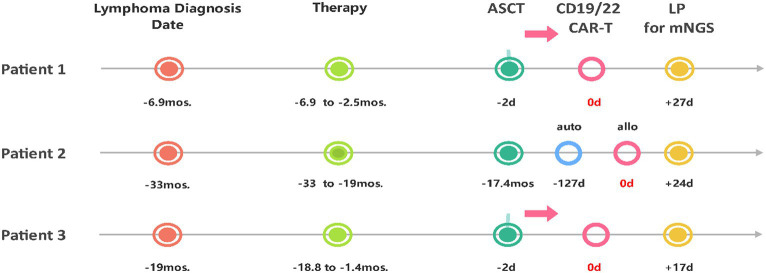
Timeline from the diagnosis of lymphoma, through the course of treatment, to the diagnosis of HHV6B encephalitis via lumbar puncture with mNGS. ASCT, Autologous Stem Cell Transplantation; auto, autologous; allo, allogeneic; LP, lumbar puncture; mos., months; d, day(s).

As shown in Table 1 and Figure 1, patient 1 was a 47-year-old male who experienced relapse after six lines of therapy. The patient received CD19/CD22 CAR-T cell cocktail therapy following autologous stem cell transplantation (ASCT). Prophylactic antiviral therapy with ganciclovir was administered 2 weeks before stem cell infusion. Treatment was complicated by grade 2 CRS and immune effector cell-associated neurotoxicity syndrome (ICANS). Engraftments were delayed until death on the 30th day after CTI, even though he was continuously administered the low-activity antiviral acyclovir (0.4 g po bid) after transplantation (13). Given the clinical course and subsequent evidence, it remains uncertain whether the death was due to ICANS or possible HHV6 encephalitis.

**Table 1:** Clinical characteristics and outcomes.

		Patient 1	Patient 2	Patient 3
Disease characteristics				
Age		47	31	50
Gender		M	F	F
Histology		DLBCL nonGCB	DLBCL nonGCB	DLBCL nonGCB
Ann Arbor stage		IIA	IIIB	IIA
IPI score		1	3	2
Prior lines of therapies (n)		6	9	2
Doses of Rituximab (n)		5	6	9
Previous Radiation		Yes	Yes	No
Previous Lenalidomide		No	Yes	No
Previous ASCT		No	Yes	No
Previous CART		No	Yes	No
Prophylaxis of antimicrobials				
Prophylaxis of	Antibacterial	Yes	Yes	Yes
	Antifungal	Yes	Yes	Yes
	Anti-*P.j.*	Yes	No	Yes
	Antiviral	Yes	No	Yes
Treatments and responses				
Conditioning		BEAM	FC	BEAM
T cells source		autologous	haploidentical	autologous
CAR T cells (doses)	CD19	5 × 10^6/Kg	9 × 10^6/Kg	4.7 × 10^6/Kg
	CD22	5 × 10^6/Kg	8 × 10^6/Kg	3.6 × 10^6/Kg
Antivirals post CTI	Acyclovir	Yes	No	No
	Ganciclovir	No	No	Yes
	Foscarnet sodium	No	No	Yes
IVIgG post CTI		Yes	Yes	Yes
Days of engraftments	Neutrophil	Delayed	24	12
	Platelet	Delayed	Delayed	8
Days of lymphopenia after CTI		30	12	29
Grade 5 CTCAE		Yes	Yes	No
Grade 3–4 CTCAE		Yes	Yes	Yes
Grade 1–2 CRS		Yes	Yes	Yes
Outcomes				
PFS (months)		NA	NA	6
OS (days)		30	24	185
Dead		Yes	Yes	No

ECOG, Eastern Cooperative Oncology Group; DLBCL, diffuse large B-cell lymphoma; IPI, international prognostic index; n, number; ASCT, autologous stem cell transplantation; CART, chimeric antigen receptor T cell; P.j., Pneumocystis jiroveci; CTI, CAR T cell infusion; CTCAE, the common terminology criteria for adverse events; CRS, cytokine release syndrome; PFS, progression-free survival (relative to CTI day); OS, overall survival (relative to CTI day). Definitions: PFS and OS deadlines until May 31, 2021. CRS was graded using the scale proposed by Lee et al. (17). CTCAE was referenced according to the criteria outlined in the Common Terminology Criteria for Adverse Events Version 5.0, as published by the U.S. Department of Health and Human Services, National Institutes of Health, National Cancer Institute. Neutrophil engraftment was defined as an absolute neutrophil count (ANC) > 500 cells/μL without medicine supports for 5 consecutive days. Platelet engraftment was defined as a platelet count > 20 × 10^9^/L without transfusion for 7 consecutive days. Lymphopenia was defined as an absolute lymphocyte count (ALC) < 300 cells/μL. Clinical characteristics that influenced the grading for each patient, as follows: Patient 1: Presented with altered mental status, incoherent speech, personality changes, involuntary limb tremors and shallow coma. Patient 2: Presented with initial loss of long-term memory and lucidity, followed by disorientation, dissociation, apathy, inability to recognize relatives, anterograde amnesia and status epilepticus. Patient 3: Presented with back pain and urinary retention, possibly indicating spinal cord involvement, and later developed altered consciousness and sudden seizures and status epilepticus.

**Figure 2:**
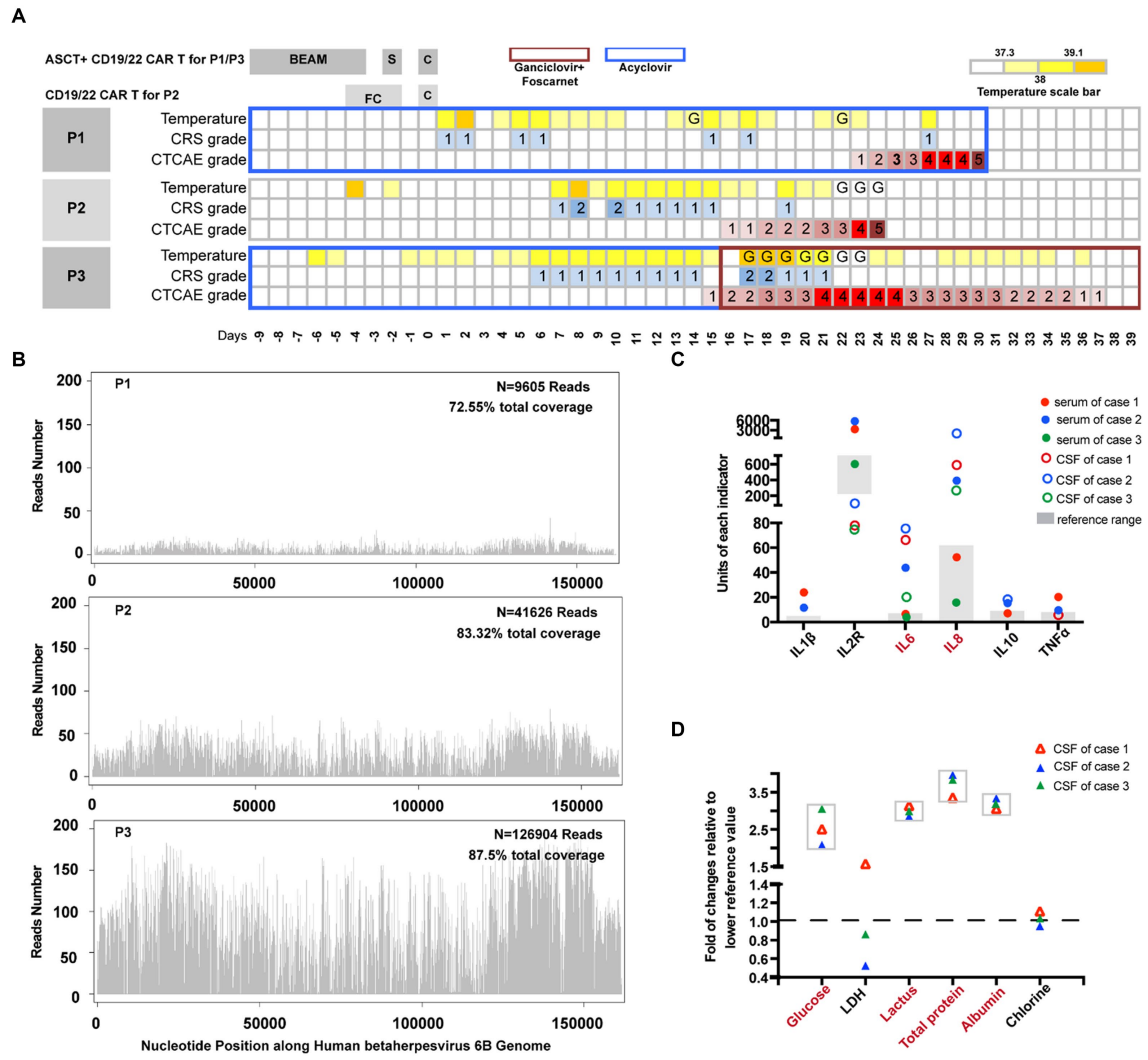
Features of clinical presentations and laboratory examinations. **(A)** Clinical presentations of HHV6 reactivation patients; S, SCI, Stem Cell Infusion; **(C)**, CTI; G, IVIgG. **(B)** mNGS of CSF samples of the patients. The identified sequence read numbers corresponding to HHV6B were 9,605, 41,626, and 126,904, with genomic coverages of 72.55, 83.32, and 87.5%, respectively. **(C)** Trends of cytokines both in serum and CSF. The units are as follows: IL2R: U/mL; IL1β, IL6, IL8, IL10, TNFα: pg./mL. **(D)** Biochemistry results of CSF.

Patient 2 was a 31-year-old female who experienced relapse after nine lines of therapy (Table 1; Figure 1), including ASCT (July 2018) and autologous CD22/CD19 CAR-T cell cocktail therapy (August 2019). She received salvage allogeneic CD22/19 CTI in the 3rd month after autologous CAR-T cell therapy. No prophylactic antiviral therapy was administered before or after allogeneic CD22/19 CTI. The CTI was complicated by grades 2 CRS and ICANS. IVIgG intravenous pulse therapy (0.4 g/kg iv for 3 days) in the late course failed to improve the outcomes (Figure 2A). Similar to Patient 1, the exact cause of death, whether ICANS or HHV6 encephalitis, remains unclear due to overlapping clinical presentations.

Patient 3 was a 50-year-old female who experienced relapse after two lines of therapy (Table 1; Figure 1). She was enrolled in a study of sequential infusion of CD22 and CD19 CAR-T cells following ASCT. She regularly received prophylactic antiviral therapy with intravenous ganciclovir for 2 weeks before autologous stem cell infusion. Subsequent therapy was complicated by grade 2 CRS and grade 4 ICANS. However, the patient’s clinical course also raised significant concerns about HHV6 infection. The patient required high-activity antiviral therapy (13), combined ganciclovir (5 mg/kg, iv, q12h), and foscarnet (60 mg/kg, iv, q12h) until clearance of HHV6B DNA in the blood, additionally with IgG infusions for 7 days at acute-onset (Figure 2A). Finally, the patient achieved CR after 7 months of follow-up, without HHV-6 reactivation.

Some typical and notable clinical details of these three patients can be summarized as follows:

All three patients presented with a fever lasting at least 2 weeks and were resistant to low-activity antiviral and other antimicrobial regimens (Figure 2A) within 1 week after CTI. All patients developed low-grade CRS (grades 1/2). Delirium and memory loss that appeared 2 weeks after CTI were the earliest mental presentations. Neurological manifestations progressed rapidly, with patients experiencing varying degrees of impaired consciousness, seizures, and coma. Back pain, lumbago, lower limb weakness, and uroschesis were observed in Patient 3, which were symptoms that may be consistent with myelitis, though they were not specific to this condition alone. Brain CT scans were performed in Patient 1 and Patient 3 when they presented with the aforementioned manifestations, and no obvious abnormalities were observed. CSF specimens were collected for pathogen detection when mental dysfunction occurred. High loads of HHV6B were detected in all CSF samples (Figure 2B) using mNGS. The identified sequence read numbers corresponding to HHV6B were 9,605, 41,626, and 126,904, with genomic coverages of 72.55, 83.32, and 87.5%, respectively. The most recent developments in molecular techniques, namely mNGS, have the potential to provide novel opportunities for the accurate investigation of infections. However, viral loads were confirmed using the most accurate and generally accepted droplet digital polymerase chain reaction (ddPCR) (18, 19). The sequences of the primers and probes used in ddPCR are listed in Supplementary Table S1. Patient 1 had an HHV-6 copy number ratio of 251.78 in blood and 503.85 in CSF. Patient 3 had 1172.41 in blood and 4694.16 in CSF. Patient 2, tested in CSF only, had a copy number ratio of 1714.69 copies/μg cell-free DNA (cfDNA). Based on HHV-6B DNA in the CSF coinciding with acute-onset altered mental status, short-term memory loss, confusion, and seizures, the diagnosis of HHV-6 encephalitis/myelitis was confirmed (15).

As shown in Figure 2C, serum cytokine levels were also analyzed for correlation with those in the CSF. A positive correlation was observed. Moreover, the concentrations of IL-6 and IL-8 were significantly higher in the CSF than in the serum. The degree of IL-8 enrichment was significant. According to a previous study on HHV6B, CSF cell counts were often unremarkable (15), whereas the fold changes in glucose, lactate, and protein levels were extremely elevated relative to the lower reference values (Figure 2D, the actual values and reference ranges for each CSF component in Supplementary Table S2). Notably, we also observed concomitant severe hyponatremia (Na + < 130 mmol/L) and/or hypernatremia (Na+ > 150 mmol/L). This suggests that it may be caused by the abnormal regulation of the central nervous system by blood sodium.

## Discussion

The increasing use of CAR T-cell therapy for the treatment of malignancies has led to improvements in the survival of patients with R/R aggressive B-cell lymphoma. However, this therapy also poses risks of infections, particularly during the early period after CAR-T cell therapy. In our observation, we identified three patients who developed acute HHV6B encephalitis/myelitis reactivation after receiving CAR-T cell therapy, and two of them developed fatal encephalitis.

Therefore, it is crucial to urgently recognize HHV-6B encephalitis/myelitis following CAR T-cell therapy due to its potential for fatality. Hematologists should recognize and distinguish it from overlapping CRS or other immunotherapy-related neurotoxicities as early as possible. However, similar clinical symptoms and onset times may present a crucial challenge in accurately diagnosing CNS dysfunction in these patients. A case report by Rebechi et al. also demonstrates a significant overlap in the clinical signs and symptoms associated with CAR-T-associated neurotoxicity and HHV-6 encephalitis (10). Handley et al. presented a scenario where the attribution of neurological symptoms and signs to ICANS could potentially lead to CNS infections being overlooked (11). The traditional diagnostic approach is particularly challenging for patients who receive CAR-T cell therapy owing to the overlapping clinical manifestations of infectious and noninfectious causes. HHV6B-associated encephalitis/myelitis may also be ignored as a CAR T therapy-related neurotoxicity due to a lack of knowledge. ICANS as a clinical entity has no diagnostic gold standard for diagnosis (17). While ICANS does have some distinctive clinical features such as apraxia or aphasia out of proportion with overall mental status, there is no definitive rule-in test to establish a diagnosis. Biomarkers such as Electroencephalography (EEG) and MRI are often normal or nonspecific in ICANS, although when positive can provide a diagnosis. Transcranial Doppler sonography (TCD) can be a helpful biomarker in the condition based on limited data. Seizure is very rare in ICANS, and while systemic inflammatory marker elevation in the form of CRS can occur preceding or concurrently with ICANS, it can also occur in isolation without inflammatory markers. Importantly, timing is a critical factor in consideration of pre-test probability for ICANS. The late onset of neurotoxic symptoms in the patients here, with lumbar puncture (LP) performed 17–27 days after transplantation, is the primary clinical factor we would use in these patients to drive diagnostics for a secondary cause of altered mental status (20, 21).

Given the limitations of traditional diagnostic methods, Rebechi et al. skillfully employed PCR technology to detect HHV6B in CSF and peripheral blood. However, they identified certain shortcomings in this approach. While HHV6B activation could be detected in the peripheral blood, it did not necessarily indicate progression to encephalitis. Additionally, they explored the potential of MRI for diagnosing HHV6B encephalitis; nevertheless, the typical radiological abnormalities associated with HHV6B encephalitis were only confirmed in one case (10). Handley et al. reported the case of a patient with refractory DLBCL treated with CAR T therapy. The patient developed CRS and ICANS on day 5 post-CTI, followed by acute bilateral lower limb weakness progressing to paralysis on day 10. Upper limb reflexes were normal but with increased tone. Based on the MRI findings, a diagnosis of ICANS-related myelitis was considered. Treatment with corticosteroids did not improve the condition, and a lumbar puncture on day 14 showed elevated protein levels. PCR tests for CMV, HSV, VZV and enterovirus on CSF were all negative. On day 16, the HHV-6 PCR on CSF was positive, and treatment with foscarnet was started. However, the patient’s condition continued to deteriorate and eventually led to death. Conventional PCR testing is limited by its inability to test for multiple viruses simultaneously and its relatively slow turnaround time, which is a challenge in urgent clinical scenarios (11). Therefore, in this case report, we propose mNGS as a novel molecular technique that offers new possibilities for the early diagnosis of HHV6B. This method is not only accurate and rapid but also provides new opportunities for investigating infections, especially in cases where the pathogen is difficult to culture. It showcases unique advantages and application prospects. It should be noted that mNGS typically offers a shorter turnaround time (within 48 h) but incurs higher costs compared to dedicated PCR. It is also important to acknowledge that these factors, as well as the cost–benefit analysis, can vary significantly across different institutions. Due to the specificity and sensitivity of the current molecular techniques, detecting HHV6B DNA in the CSF is considered sufficient for the diagnosing active CNS, regardless of the level of viremia (22–24). However, detectable viremia is generally considered a hallmark of systemic active infection (25) and is a major risk factor for HHV-6B encephalitis (26). This indicates that early recognition of HHV6B viremia using mNGS or other techniques may allow for an earlier warning of HHV6B encephalitis/myelitis in this immunocompromised setting. Consequently, patients can undergo immediate intervention for early symptoms involving the central nervous system, reducing infection-associated mortality.

To date, prophylactic or preemptive anti-HHV-6 therapy is not recommended for preventing HHV6B reactivation or encephalitis after HSCT (27–29) on the following considerations: First, antiviral drug selection should consider side effects, including the nephrotoxic (foscarnet) and myelosuppressive (ganciclovir) properties of the available agents. There is moderate evidence of a causal relationship between HHV6B and myelosuppression and allograft failure (30–32). Once a side effect occurs, this can lead to a vicious cycle. Second, previous data have demonstrated that preemptive ganciclovir or foscarnet did not significantly reduce the risk of HHV-6B encephalitis (33, 34), although the incidence and titer of HHV-6B DNA in the plasma were dramatically lower in patients receiving prophylactic antivirals (28, 29). In transplant recipients with clinical presentations of encephalitis/myelitis without other evident causes, empirical treatment for HHV6B should be considered (35). This seems to be in accordance with the outcome of Patient 3 in our study. For HHV6B encephalitis/myelitis, at least 3 weeks of antiviral therapy should be administered until clearance of HHV-6 DNA from the blood and, if feasible, CSF (35).

Our report aims to remind clinicians of two critical points: First, not to overlook the possibility of fatal HHV-6 CNS infection following CAR-T therapy, especially in the presence of delirium or sleep deprivation. Second, the importance of mNGS for early and rapid pathogen screening, combined with ddPCR for confirmation and clinical correlation, is critical for early diagnosis and intervention, potentially saving lives. This is exemplified in our cases, where patients 1 and 2 had worse outcomes due to delayed detection, while Patient 3, benefiting from our accumulated experience, had a better prognosis with early detection and treatment.

Although our study has important implications, it has some limitations. Remarkably, the small sample size did not allow us to analyze the risk factors or optimal therapeutic strategies. Future studies are needed to elucidate the potential risk factors for reactivation of HHV-6 in associated complications of post-CAR-T cell-based therapy, especially those involving the central nervous system, and the exploitation of effective and safe strategies to mitigate HHV-6 reactivation warrants continued attention.

## Statement

The Common Terminology Criteria for Adverse Events, version 5.0 (CTCAE v5.0), created by the National Cancer Institute, is a versatile tool in clinical research, providing a comprehensive framework for grading a wide range of adverse events, including neurological conditions like encephalitis. In contrast, the ASTCT (American Society for Transplantation and Cellular Therapy) criteria, while highly specialized, are specifically tailored for CRS and neurotoxicity in Immune Effector Cell therapies, offering a more focused approach compared to the broader applicability of the CTCAE. Due to the initial diagnosis of suspicious ICANS being revised to encephalitis after mNGS evaluation, we graded the neurological symptoms using CTCAE v5.0 instead of ASTCT criteria, ensuring a more appropriate and comprehensive grading system for the patient’s diagnosed condition.

## Data availability statement

The original contributions presented in the study are included in the article/Supplementary material, further inquiries can be directed to the corresponding authors.

## Ethics statement

The studies involving humans were approved by the Institutional Review Board of Tongji Hospital, Tongji Medical College, Huazhong University of Science and Technology. The studies were conducted in accordance with the local legislation and institutional requirements. The participants provided their written informed consent to participate in this study. Written informed consent was obtained from the individual(s) for the publication of any potentially identifiable images or data included in this article.

## Author contributions

NL: Data curation, Writing – original draft. RZ: Data curation, Writing – original draft. JW: Writing – original draft. XZ: Writing – original draft. FM: Writing – original draft. YC: Writing – original draft, Data curation, Funding acquisition, Writing – review & editing. GW: Data curation, Funding acquisition, Writing – original draft, Writing – review & editing. YY: Data curation, Funding acquisition, Writing – original draft, Writing – review & editing.
