# Supplementary material for: Case report: Acute HHV6B encephalitis/myelitis post CAR-T cell therapy in patients with relapsed/refractory aggressive B-cell lymphoma
Source: Front Neurol. 2024 Feb 29;15:1334000. doi: 10.3389/fneur.2024.1334000 (PMC10937551; doi:10.3389/fneur.2024.1334000)
Supplement: Supplementary file 1 [file Data_Sheet_1.PDF]

Supplemental Tables

Table S1. The Sequences of the Primers and Probes Used in ddPCR

Genes	Primers/Probes	Sequences
HHV-6B	Forward primer	5'-TCCATTGTTTGATTGATTTCCGTAT-3'
	Reverse primer	5'-AACGCGGGATGTTCTATTGG-3'
	Probe	5'-FAM-CTTGAGCTTGTAGATAAT-3'
POP4	Forward primer	5'-GGCGGTGGTCCTGGAGTACT-3'
	Reverse primer	5'-AGAGGCCTTTGGCTTTCTTCTT-3'
	Probe	5'-VIC-ACCCGCCACAAGC-3'

Table S2. Biochemistry results of CSF

Parameter	Patient 1	Patient 2	Patient 3	Reference range
Glucose (mmol/L)	5.57	4.62	6.68	2.22-3.89
LDH (U/L)	63	21	33	<40
Lactate (mmol/L)	3.45	3.13	3.33	1.10-2.40
Total Protein (mg/L)	505	595	568	150-450
Albumin (mg/L)	306	334	340	100-300
Chloride (mmol/L)	132.9	114.4	122.2	120-130
Nucleated Cell Count (×10^6/L)	2	4	0	0-8

Supplemental Methods

mNGS Methodology

Plasma sample: Sample Processing and DNA Extraction

Volume of 3 mL of blood were drawn from patients, placed in blood collection tube and stored at room temperature for 3-5 minutes before plasma separation and centrifuged at 4,000 rpm for 10 min at 4°C within 8 h of collection. Plasma samples were transferred to new sterile tubes. DNA was extracted from 300 μL of plasma using the TIANamp Micro DNA Kit (DP316, TIANGEN BIOTECH , Beijing, China) following the manufacturer's operational manual. The extracted DNA specimens were used for the construction of DNA libraries[1].

Respiratory and pleural fluid samples：Sample Processing and DNA Extraction

1.5-3mL sputum/BALF/pleural fluid and other samples from patient was collected according to standard procedures. Saponin was added to 0.45mL sample at a final concentration of 0.025%. Then the sample was fully vortexed for 15s and incubated for 5 min at 25 °C. 75 μL was added for dehosting process. The sample was fully vortexed for 15s and incubated at 37 °C for 10 min. Then the sample was centrifuged at 18,000 g for 5 min and ~70-80μL were remained at the bottom after removal of 450μL supernatant. 800μL PBS was added to the tube and fully vortexed. After centrifugation at 18,000g for 5 minutes, 800μL supernatant was discarded and ~70-80μL were remained at the bottom. Add 370μL TE-buffer to the tube, followed by shaking. Then 7.2μL lysozyme was added for wall-breaking reaction. 250μL 0.5mm glass bead were attached to a

horizontal platform on a vortex mixer and agitated vigorously at 2800-3200 rpm for 30 min. 0.3mL sample was separated into a new 1.5mL microcentrifuge tube and DNA was extracted using the TIANamp Micro DNA Kit (DP316, TIANGEN BIOTECH) according to the manufacturer's recommendation.

Body fluid sample: Sample Processing and DNA Extraction

1.5-3mL CSF and other Body fluid samples from patient was collected according to standard procedures. 1.5mL microcentrifuge tube with 0.6mL sample and 250μL 0.5mm glass bead were attached to a horizontal platform on a vortex mixer and agitated vigorously at 2800-3200 rpm for 30 min. Then 7.2μL lysozyme was added for wall-breaking reaction. 0.3mL sample was separated into a new 1.5mL microcentrifuge tube and DNA was extracted using the TIANamp Micro DNA Kit (DP316, TIANGEN BIOTECH) according to the manufacturer's recommendation.

Tissue samples: Sample Processing and DNA Extraction

Tissue blocks the size of soybeans were collected according to the standard sample collection procedure. The tissue blocks, 600μL of lysis buffer and 250μL 0.5mm glass beads were attached to a horizontal platform on a vortex mixer and agitated vigorously at 2800-3200 rpm for 30 min. Then 7.2μL lysozyme was added for wall-breaking reaction. 0.3mL sample was separated into a new 1.5mL microcentrifuge tube and DNA was extracted using the TIANamp Micro DNA Kit (DP316, TIANGEN BIOTECH) according to the manufacturer's recommendation.

Construction of DNA libraries and Sequencing

Then, DNA libraries were constructed through DNA-fragmentation, end-repair, adapter-ligation and PCR amplification. Agilent 2100 was used for quality control of the DNA libraries. Quality qualified libraries were pooled, DNA Nanoball (DNB) was made and sequenced by BGISEQ-50 /MGISEQ-2000 platform[2].

Bioinformatic analysis

High-quality sequencing data were generated by removing low-quality reads, followed by computational substraction of human host sequences mapped to the human reference genome (hg19) using Burrows-Wheeler Alignment[3]. The remaining data by removal of low-complexity reads were classified by simultaneously aligning to Pathogens metagenomics Database (PMDB), consisting of bacteria, fungi, viruses and parasites. The classification reference databases were downloaded from NCBI (ftp://ftp.ncbi.nlm.nih.gov/genomes/).

Pathogen detection thresholds

Criteria for detecting the presence of pathogens were set to minimize false positives due to microbial contamination. In the case of viruses, the threshold was the detection of non-overlapping reads from three or more different genomic regions[4]. A virus type (DNA or RNA) was classified as 'detected' if it met this criterion and as 'not detected' if it did not. For bacteria and fungi, the threshold was based on the ratio of RPTM values between the sample and the no-template control (NTC), with the minimum RPTM of the NTC set at one. This approach accounted for low levels of microbial contamination by normalizing pathogen-specific reads relative to the NTC. The NTC, consisting of

sterile water, was run alongside the clinical test samples to monitor contamination during the mNGS process.

ddPCR Methodology

Sample collection and cfDNA extraction

Peripheral blood (PB) samples (10 mL) were collected after informed consent in EDTA-K2 anticoagulant tubes with immediate mixing to prevent clotting. Cerebrospinal fluid (CSF) samples (5 mL) were also collected and stored at 4°C, then transferred to -80°C within 8 hours. Cell-free DNA (cfDNA) was extracted using a QIAamp Circulating Nucleic Acid Kit, quantified using a Qubit Fluorometer 3.0, and stored at -20°C for ddPCR.

Primer and probe design and plasmid generation

Specific primers and probes for HHV-6B and the human RNase P protein POP4 gene were designed and synthesized, labelled with FAM and VIC fluorophores (Table S2). A plasmid containing the HHV-6B sequence was constructed, linearized and used to generate standards for ddPCR validation.

Digital droplet PCR for HHV-6B detection

ddPCR was performed on a 20 μL reaction mixture using the QX200 Droplet Digital PCR System. Analysis was performed on the QX200 Droplet Reader using Quantasoft software, with thresholds set manually based on negative controls. Droplet positivity was determined by fluorescence intensity and cfDNA copy numbers were reported as copies/μL DNA and then converted to copies/μg DNA.

Supplemental References：

[1] Y. Long, Y. Zhang, Y. Gong, R. Sun, L. Su, X. Lin, A. Shen, J. Zhou, Z. Caiji, X. Wang, D. Li, H. Wu, and H. Tan, Diagnosis of Sepsis with Cell-free DNA by Next-Generation Sequencing Technology in ICU Patients. Arch Med Res 47 (2016) 365-371.

[2] Y.J. Jeon, Y. Zhou, Y. Li, Q. Guo, J. Chen, S. Quan, A. Zhang, H. Zheng, X. Zhu, J. Lin, H. Xu, A. Wu, S.G. Park, B.C. Kim, H.J. Joo, H. Chen, and J. Bhak, The feasibility study of non-invasive fetal trisomy 18 and 21 detection with semiconductor sequencing platform. PLoS One 9 (2014) e110240.

[3] H. Li, and R. Durbin, Fast and accurate short read alignment with Burrows-Wheeler transform. Bioinformatics 25 (2009) 1754-60.

[4] T.A. Blauwkamp, S. Thair, M.J. Rosen, L. Blair, M.S. Lindner, I.D. Vilfan, T. Kawli, F.C. Christians, S. Venkatasubrahmanyam, G.D. Wall, A. Cheung, Z.N. Rogers, G. Meshulam-Simon, L. Huijse, S. Balakrishnan, J.V. Quinn, D. Hollemon, D.K. Hong, M.L. Vaughn, M. Kertesz, S. Bercovici, J.C. Wilber, and S. Yang, Analytical and clinical validation of a microbial cell-free DNA sequencing test for infectious disease. Nat Microbiol 4 (2019) 663-674.
